# Acute lung injury and the acute respiratory distress syndrome in the injured patient

**DOI:** 10.1186/1757-7241-20-54

**Published:** 2012-08-10

**Authors:** Magdalena Bakowitz, Brandon Bruns, Maureen McCunn

**Affiliations:** 1 Department of Anesthesiology & Critical Care, University of Pennsylvania, 3400 Spruce Street, Philadelphia, PA, 19104, USA; 2 Division of Traumatology, Surgical Critical Care and Emergency Surgery, University of Pennsylvania, 3400 Spruce Street, Philadelphia, PA, 19104, USA; 3 Department of Anesthesiology & Critical Care, University of Pennsylvania, 3400 Spruce Street, Philadelphia, PA, 19104, USA

**Keywords:** Lung injury, Acute lung injury (ALI), Acute respiratory distress syndrome (ARDS), Trauma, Injury, Prone positioning, Extracorporeal membrane oxygenation (ECMO), Rib plating, Rib open reduction internal fixation, Flail chest, High-frequency oscillatory ventilation (HFOV), Airway pressure release ventilation (APRV)

## Abstract

Acute lung injury and acute respiratory distress syndrome are clinical entities of multi-factorial origin frequently seen in traumatically injured patients requiring intensive care. We performed an unsystematic search using PubMed and the Cochrane Database of Systematic Reviews up to January 2012. The purpose of this article is to review recent evidence for the pathophysiology and the management of acute lung injury/acute respiratory distress syndrome in the critically injured patient. Lung protective ventilation remains the most beneficial therapy. Future trials should compare intervention groups to controls receiving lung protective ventilation, and focus on relevant outcome measures such as duration of mechanical ventilation, length of intensive care unit stay, and mortality.

## Introduction

The Acute Respiratory Distress Syndrome (ARDS) was originally described in 1967 by Ashbaugh and Levine [1], and despite advances in diagnosis and treatment of the condition over the ensuing forty plus years, it remains a significant contributing factor to morbidity in the traumatically injured patient [2]. Survivors of ARDS have a lower functional ability and lower than normal health related quality of life two years after hospital discharge [3]. The treatment and rehabilitation of ARDS carries with it a great societal cost, and thus it remains a disease process of the utmost importance [4]. This review focuses on ALI and ARDS in the traumatically injured patient. The current definitions of acute lung injury (ALI) and acute respiratory distress syndrome (ARDS) stem from the American-European Consensus Conference, first published in 1994 [5]. The diagnosis of ALI is a clinical one and requires the presence of an acute onset, a partial pressure of arterial oxygen (PaO_2_) in mm Hg to fraction of inspired oxygen (FiO_2_) ratio (P/F ratio) of less than 300 if measured in mm Hg, or less than 40 if measured in kPa, bilateral infiltrates on chest radiograph, and the lack of evidence of cardiogenic pulmonary edema or a pulmonary artery occlusion less than or equal to 18 mm Hg or 2.4 kPa. The definition for ARDS is the same as above, with the exception that the P/F ratio is less than 200 if measured in mm Hg (less than 27 if measured in kPa), or less than 150 if measured in mm Hg (less than 23 if measured in kPa) for severe ARDS. The definitions have been challenged since the discovery that higher FiO_2_ and positive end-expiratory pressure (PEEP) levels might convert patients who previously met ARDS criteria to be classified as ALI cases by P/F ratio, and patients who previously would have been classified as ALI to carry neither lung injury diagnosis [6,7].

### Incidence and epidemiology

While sepsis is the most common risk factor for ALI (representing one third of cases), trauma has been identified to constitute at least 7% of cases [8]. When first studied, the incidence of ARDS did not differ between patients with blunt and penetrating mechanisms of injury, with comparable and declining mortality in both groups [9]. Over the last decade, mortality rates have continued to decrease in the general population of patients with ALI/ARDS [10,11]. The mortality of trauma-associated ALI is estimated at 24% [8,12,13]. For trauma patients treated at hospitals participating in ARDS Network trials, the 60-day mortality rate is exceptionally low at 10%, but has not seen any further decrease in recent years [11]. Ciesla *et al.*[14] however reported an encouraging decrease in the progression of ALI to ARDS and multiple organ failure (MOF) in a large single center trauma cohort. This must be considered in light of changing therapies for sepsis, cardiovascular failure, and renal replacement therapies.

A 2002 examination of ALI utilizing data from the United States showed an estimated 190,000 cases and 74,000 deaths from ALI on a yearly basis [8]. A more recent analysis of the National Trauma Databank shows the incidence of trauma-related ARDS to be 6.5% of traumatically injured patients requiring mechanical ventilation for greater than 48 hours [15]. Of patients developing ARDS, the rate of pneumonia approached 50% with crude mortality of 19%. Patients spent on average 20 days on the ventilator, 22 days in the intensive care unit (ICU), and 32 days in the hospital. Total charges incurred during hospitalization were upwards of $244,000 [15].

A 2006 retrospective review of trauma ICU data at the University of Southern California showed an overall complication rate of 43% in patients with ARDS. Complications included pneumonia, deep venous thrombosis, pulmonary embolism, acute renal failure, and disseminated intravascular coagulopathy. Additionally, ARDS patients had longer hospital stays than similarly matched controls, longer ICU stays, and higher hospital charges ($267,037 vs. $136,680) [16]. Despite these differences, multiple investigators have shown no difference in mortality between ARDS patients and matched controls [16,17].

A review of blunt traumatic brain injury (TBI) patients with a head abbreviated injury score (AIS) greater than or equal to 4 showed a 7.7% incidence of ARDS. The incidence of mortality was similar in TBI patients with and without ARDS (50% vs. 51.8 %), no significant difference in regards to discharge functional capacity between the two groups. Again, patients in the TBI + ARDS group had more complications (pneumonia, disseminated intravascular coagulation, sepsis), more time spent in the ICU, and more total hospital days [18].

ARDS following trauma is different in many respects from ARDS related to other causes. Patients with ARDS after injury tend to be younger and have fewer comorbid medical conditions [19]. Endothelial activation factor levels are lower in patients with ARDS related to trauma as opposed to septic insults [20-22]. Following cellular injury, endothelial cells can become activated, a principal mechanism in the complex pathologic events that result in ARDS. Although there is no uniform agreement on the definition of endothelial activation, inflammatory conditions lead to transcription of mRNA, altered synthesis of proteins, and a change in phenotype or function in response to stimuli from the environment [23]. Specifically, subgroup analysis of ARDS Network data has shown significantly lower levels of serum biomarkers (von Willebrand factor antigen [vWF], surfactant protein D [SP-D], tumor necrosis factor receptor-1 [TNFr-1], and intercellular adhesion molecule-1 [ICAM-1], associated with poor outcome in trauma patients [19]. Moreover, the mortality of ARDS in victims of trauma is lower than those with ARDS from other causes after adjusting for baseline differences [17].

Independent risk factors for the development of ARDS after blunt trauma are those with an injury severity score (ISS) > 25, the presence of pulmonary contusion, a large transfusion requirement, hypotension on admission, and age > 65 years [24]. Furthermore, investigators have shown that increasing Acute Physiology and Chronic Health Evaluation II (APACHE II) scores along with duration of mechanical ventilation are independent risk factors for the development of ARDS after trauma [25]. Some have shown specific injury patterns, such as long bone fracture and chest injury, to be independently associated [26]. Some have suggested that post-traumatic ARDS comes in two distinct forms, early (< 48 hours) or late (> 48 hours). Those developing early ARDS are characterized by a higher rate of penetrating injury, a lower admission base deficit, greater 48-hour transfusion requirement, more fluid administration in the first 5-day period, and a lower P/F ratio on presentation; namely patients presenting in hemorrhagic shock. Patients in the late ARDS group tended to be older and had suffered a bout of pneumonia during their hospitalization [27]. The mortality rate is similar between both early and late groups [26,27].

### Pathophysiology of ARDS

**Figure 1:**
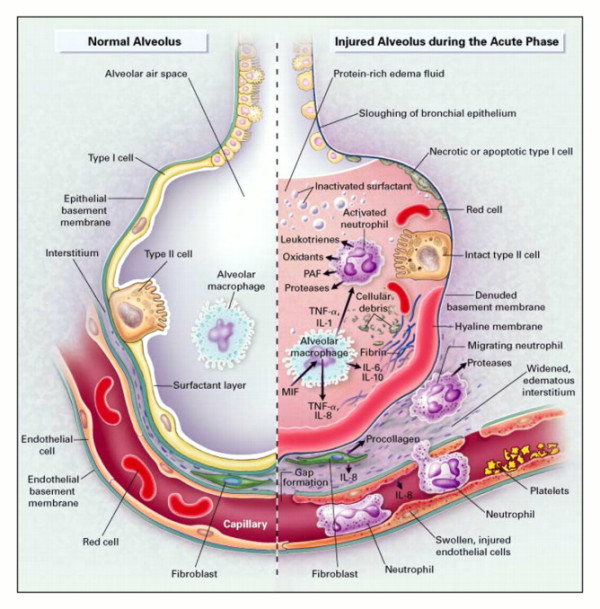
**The normal alveolus (Left-Hand Side) and the injured alveolus in the acute phase of acute lung injury and the acute respiratory distress syndrome (Right-Hand Side).** Reproduced with permission, fee paid.

**Figure 2:**
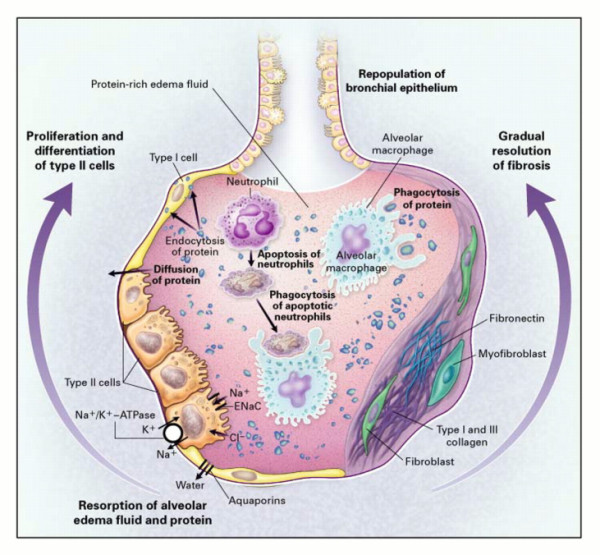
**Mechanisms important in the resolution of acute lung injury and the acute respiratory distress syndrome.** Reproduced with permission, fee paid.

**Figure 3:**
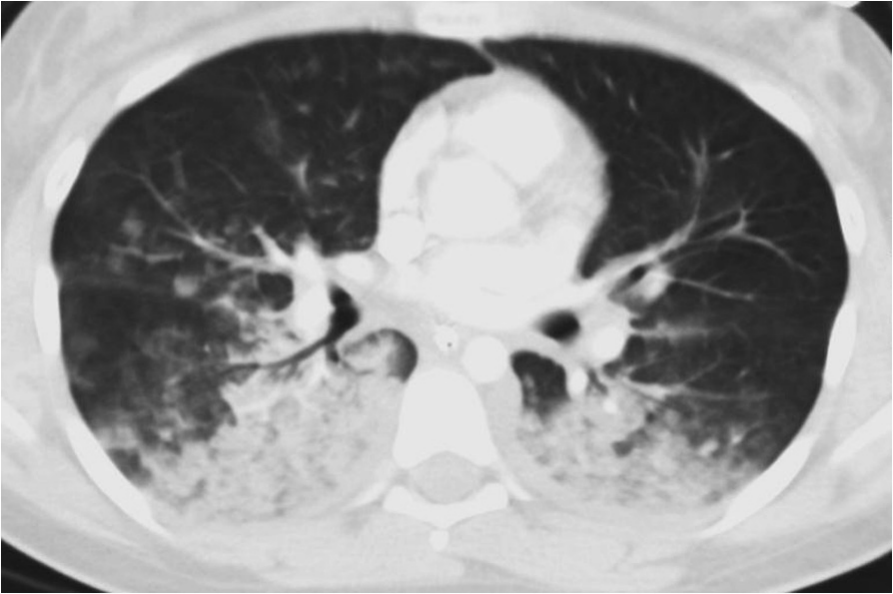
**CT scan of same patient several hours after admission.** Patient had undergone craniotomy for evacuation of intracranial hemorrhage. Intracranial pressure was not elevated post-operatively. (Atelectasis and consolidation is heterogeneous at dependent bases).

**Figure 4:**
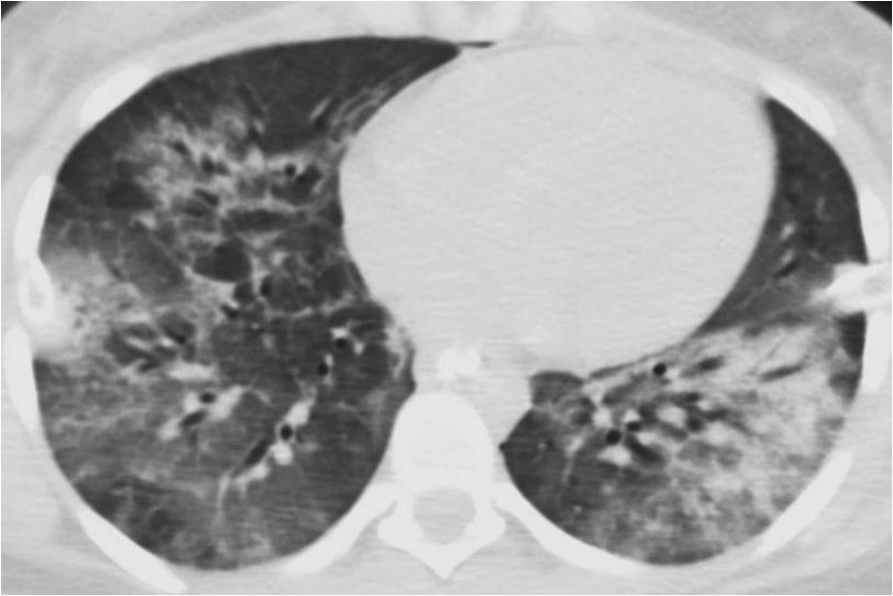
**Same patient on hospital day 9, following decannulation from ECMO.** Consolidation is homogeneous and diffuse.

ARDS is a progressive clinical condition in which the early phases of the disease are marked by dyspnea and hypoxemia with the appearance of radiographic infiltrates on chest radiography [28]. The development of ARDS is typically secondary to either direct lung injury (pneumonia, pulmonary contusion, inhalation injury) or indirect lung injury (overwhelming sepsis, transfusion, pancreatitis) [28] (Table 1). The clinical picture is a manifestation of the alveolar degradation and flooding with protein rich material and cellular debris with subsequent increases in pulmonary vascular resistance (Figure 1) [29]. A complex array of endothelial injury, epithelial injury, neutrophil-mediated damage, cytokine-mediated inflammation and injury, oxidant-mediated injury, ventilator-induced lung injury, and dysregulation of the coagulation and fibrinolytic pathways are all implicated in the development of ARDS [28,29]. A pro-inflammatory milieu emerges in the pulmonary environment, thus leading to direct parenchymal injury and clinical deterioration (Figure 2) [29]. Severe traumatic injury is the epitome of the pro-inflammatory state, and thus ARDS is seen with increased incidence in the traumatically injured patient. ARDS may be heterogeneous and dependent, or homogeneous and diffuse, as seen in Figures 3 and 4.

**Table 1:** Risk factors for ALI/ARDS

**Primary**	**Secondary**
Aspiration	Transfusion-associated lung injury (TRALI)
Contusion	Pancreatitis
Pneumonia	Sepsis
Inhalation Injury	Traumatic Brain Injury (TBI)
Ventilator-Induced Lung Injury (VILI)	

### Transfusion practices

Severely injured trauma patients requiring blood transfusion deserve special mention. Evidence clearly illustrates that early transfusion of packed red blood cells (PRBCs) is an independent predictor of ARDS and increases with increasing units of transfused blood [30,31]. Fresh frozen plasma (FFP) has also been independently associated with a greater risk of developing ARDS, whereas platelets and cryoprecipitate were not [32]. Pre-storage leukoreduction has been attempted in an effort to minimize the pro-inflammatory effects of residual leukocyte contamination of stored PRBCs, with the hopes of decreasing post-transfusion ARDS rates. However, randomized controlled trials have failed to show any difference in the risk of ALI or ARDS in patients receiving leukoreduced versus standard PRBCs at 28 days [33].

The effect of ABO-identical versus ABO-compatible non-identical plasma has also been explored. No difference in mortality was noted in the identical versus compatible groups. However, patients receiving ABO-compatible non-identical plasma had higher complication rates and significantly higher rates of ARDS and sepsis. Furthermore, the rates of ARDS and sepsis increased as more units of ABO-compatible non-identical plasma was transfused [34].

### Pulmonary contusion

As previously mentioned, pulmonary contusion is an independent risk factor for the development of ARDS after trauma [35] and as such much attention has been paid to quantification of the degree of pulmonary contusion and its ability to alert the physician to future respiratory compromise.

**Figure 5:**
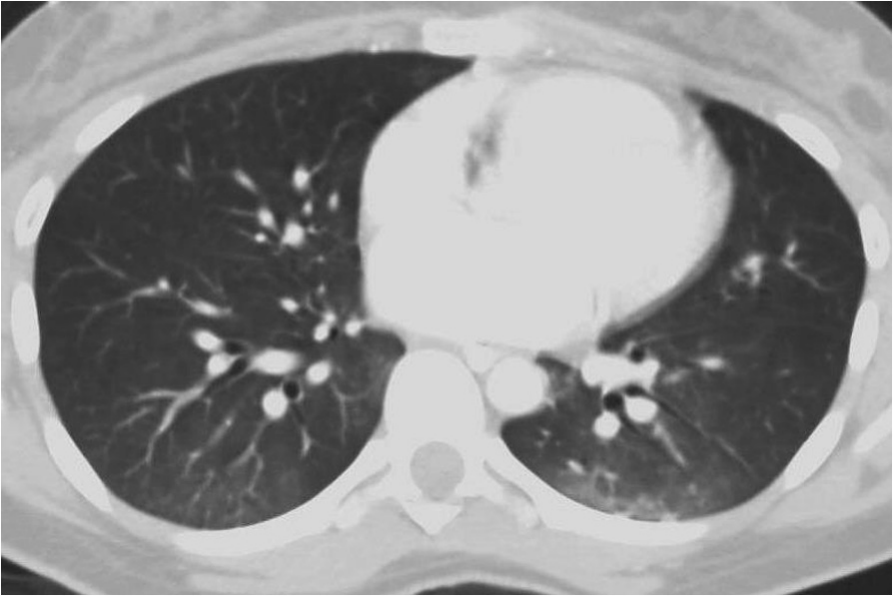
Admission CT scan of 18 year-old female with severe TBI (no pulmonary injuries identified).

**Figure 6:**
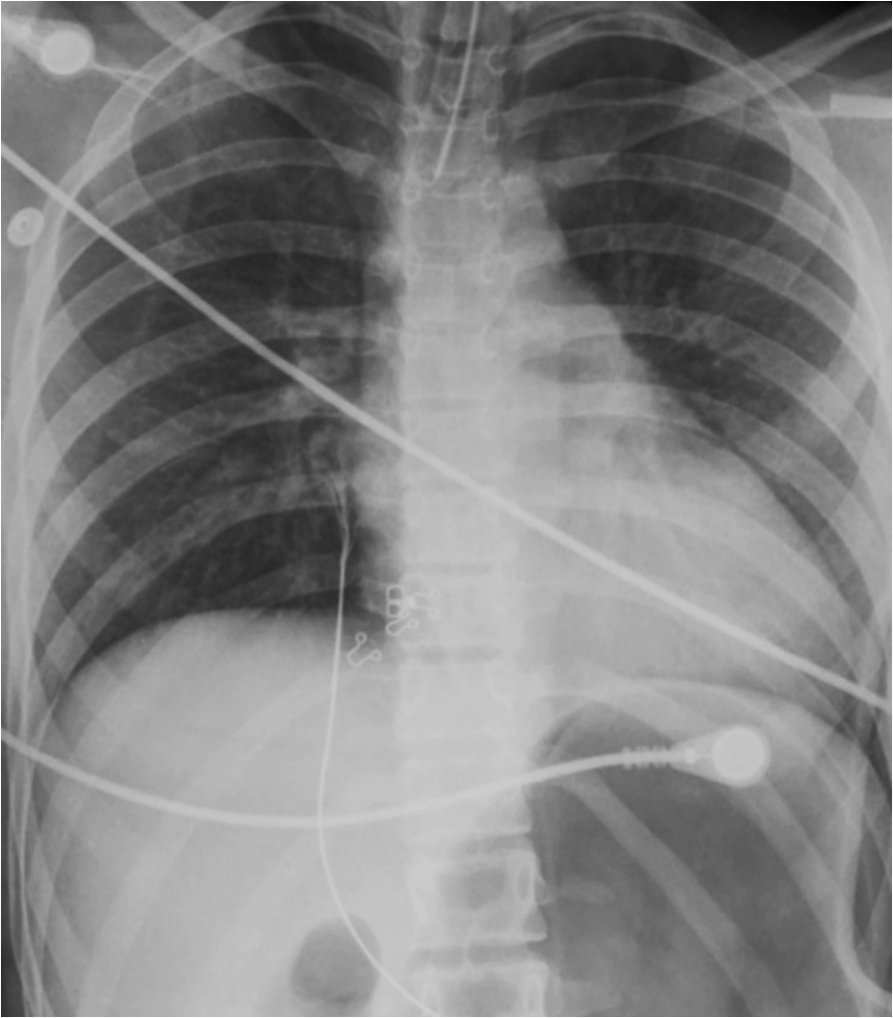
Corresponding admission CXR of 18 year-old female with severe TBI.

Some have utilized three-dimensional computed tomography in all patients with significant thoracic injury (Figures 5 and 6). These authors showed that as the pulmonary contusion percentage exceeded 20% of total lung parenchyma, a sharp increase in the incidence of ARDS occurred [35-37]. Some suggest that this could potentially be utilized as a screening tool and enable appropriate lung protective strategies early in the patient’s course. (See further discussion of pulmonary contusions under “Treatment”).

### Race and gender differences

Longitudinal epidemiologic studies have shown consistent differences in mortality amongst ARDS patients as a group. Males with ARDS have a persistently higher mortality rate than females with ARDS. Data would also suggest that African-American males with ARDS have a higher mortality rate than males of other racial backgrounds. Similarly, females of African-American race have a higher ARDS mortality rate than females of other racial backgrounds [22].

When examining ARDS after traumatic injury, investigators have shown that females are more likely to develop ARDS than their male counterparts. Despite the higher percentage of ARDS cases in females, the mortality between female and male patients with ARDS after traumatic injury does not appear to differ [38]. This data supports the concept of sex hormones, specifically estrogens, having immunologic properties enabling the development of lung injury. For both male and female patients, ARDS increases in incidence with increasing estradiol levels. Additionally, for both male and female patients, ARDS declines with increasing testosterone levels [38]. A recent study by the National Institute of Health in the United States brought awareness to a potential treatment variability [39]. Women received lung protective ventilation less frequently than men. However, after adjustment for height and severity of illness, this difference was no longer detectable.

### Nutrition

The pro-inflammatory environment of the pulmonary parenchyma in ARDS patients has led some investigators to examine the effects of enteral nutrition enriched with fish oils and borage oils containing high levels of antioxidants. Early studies suggested a favorable effect on oxygenation, number of ventilator and ICU days, and a lower incidence of new organ failure [40]. These findings have been disproven by the recent OMEGA study [41], which was stopped early for futility. Twice-daily enteral supplementation of n-3 fatty acids, γ-linolenic acid, and antioxidants did not improve the number of ventilator-free days. Not all patients are able to tolerate enteral feeds after traumatic injury, thus leading some to examine the effects of total parenteral nutrition (TPN). Ventilated trauma patients receiving TPN were retrospectively analyzed over a 6-year period. TPN was independently associated with the development of late ARDS [42]. These data would suggest that the inflammatory modulation properties of the nutritional source be carefully considered in the patient at risk for ARDS.

### Treatment

A number of management strategies relevant to acute lung injury with an emphasis on prevention of further deterioration are discussed below (Table 2).

**Table 2:** A continuum of treatment options with at times inconsistent evidence (see text for references)

**Proven benefit**	**Suggested benefit in select patients**	**Indeterminate benefit**	**Suggested as not harmful**	**No benefit**
Low tidal volumes of 6 ml/kg predicted body weight	Fluid restriction	Recruitment maneuvers	Higher PEEP	Surfactant
PEEP ≥ 5 cm H_2_O	Incentive spirometry	High-frequency oscillatory ventilation		Prostaglandin E1
Plateau pressures ≤ 30 cm H_2_O	Patient-initiated positive airway pressure therapy	Airway pressure release ventilation		N-acetylcysteine
	Non-invasive positive pressure ventilation	Steroids		
	Early use of neuromuscular blocking agents			
	Extracorporeal membrane oxygenation			
	Rib fracture fixation			
	Prone positioning			

It is common practice to resuscitate the trauma patient in shock with intravenous fluids even at the expense of worsening pulmonary edema. A fluid-conservative management strategy is recommended in the absence of hypotension or vasopressor requirement based on a randomized-controlled trial in medical and surgical ICU patients [43]. While this study did not measure a lower mortality rate, it also did not increase non-pulmonary organ failure. Instead, fluid restriction shortened the duration of mechanical ventilation and the length of intensive care unit stay. No randomized controlled studies exist that provide sufficient evidence to guide fluid management specific to the trauma population [44].

Pulmonary contusions can evolve over several days. Therefore, goals in the initial period are to prevent atelectasis and derecruitment (as seen in the patient in Figure 3 and 4). Incentive spirometry and patient initiated positive airway pressure therapy have been shown to decrease mechanical ventilator-dependent days, lengths of stay, infectious morbidity, and mortality in awake and cooperative patients with rib fractures assigned to a multidisciplinary pathway [45]. There is no evidence to draw conclusions on whether recruitment maneuvers independently reduce mortality or length of ventilation in patients with ALI or ARDS [46].

A retrospective review of adults with blunt traumatic pulmonary contusion in an Australian center demonstrated that noninvasive positive pressure ventilation (NIPPV) was successfully used to avoid intubation in a set of trauma patients deemed clinically appropriate despite severe pulmonary contusions and who met criteria for ARDS by P/F ratio [47]. Of note, NIPPV was combined with a multi-modal analgesia regimen including epidural analgesia, followed by a combination of intravenous patient-controlled analgesia, non-steroidal anti-inflammatory drugs and acetaminophen. More recently, a small randomized-controlled trial in patients with thoracic trauma came to the same conclusion in regard to the use of NIPPV to avoid intubation [48]. Patients who develop frank respiratory failure (hypoxia, hypercarbia, increased work of breathing) should be intubated and mechanically ventilated without inappropriate delay. Other indications for intubation are the need for airway protection, combativeness, cardiovascular instability, to facilitate imaging and procedures, and anticipated pulmonary deterioration. Following two landmark randomized controlled trials that demonstrated a reduced mortality in patients ventilated with small volumes and low plateau pressures [49,50], small tidal volume ventilation and the use of PEEP has been accepted to maintain alveolar recruitment and oxygenation [51]. Borges *et al.* showed that hypoxemia can be reversed and the lung fully recruited in early ARDS [52].

A well-designed trial demonstrated that in patients ventilated with low tidal volumes, higher levels of PEEP in combination with recruitment maneuvers did neither result in a difference in hospital mortality nor in the rate of barotrauma when compared to conventional levels of PEEP [53]. The optimal level of PEEP is still unknown [54,55]. While PEEP usually aids in recruitment of alveoli, ventilation/perfusion mismatch can be exacerbated with ventilator pressures in unilateral lung injury as blood flow is directed away from the more compliant lung to the injured lung [56]. Patients with unilateral pulmonary contusions can progress to a clinical picture consistent with ARDS without meeting the formal criterion of bilateral infiltrates on chest x-ray for ARDS [5]. Posttraumatic patients at risk for or who develop ARDS should be ventilated with low tidal volumes according to ARDS Network guidelines as non-traumatized patients are.

High-frequency oscillatory ventilation avoids repeated opening and closing of lung areas by delivering cycling pressures above and below a high mean airway pressure while maintaining lung recruitment and while creating small tidal volumes of 1-4 ml/kg at a minimum of 60 times per minute (1 Hz). A systematic review and meta-analysis found that high frequency oscillation ventilation as an initial ventilation mode for ALI/ARDS suggests a reduced hospital and 30-day mortality compared to patients who were treated with conventional mechanical ventilation, though this evidence is not strong [57].

Airway pressure release ventilation (APRV) is the continuous administration of positive airway pressure (CPAP) to achieve recruitment with intermittent airway pressure releases to allow CO_2_ clearance. Patients can breathe at any point while remaining at a higher mean airway pressures [58]. It is similar to inverse ratio pressure control ventilation (IRV) with the added benefit of spontaneous breathing, and without the degree of sedation or muscle relaxation necessary for IRV [59]. APRV is an alternative mode of ventilation in patients with ALI/ARDS, but has not shown improved outcomes in mortality in large trials. It has yet to be directly compared to ventilation following the ARDS Network protocol. In a recent trial conducted by Roy *et al.*[60], Yorkshire pigs sustained ischemia-reperfusion injury by clamping of the superior mesenteric artery plus induced peritoneal sepsis. The animals that were immediately randomized to APRV did not develop ARDS. A major limitation of this study is that the control group was ventilated with tidal volumes of 10 ml/kg instead of the current low-volume ventilation standard per ARDSnet guidelines. The pig study might have had a negative result, had the researchers used a tidal volume of 6 ml/kg in their control group.

Neuromuscular blocking agents have been thought to aid in faster achievement of targeted lung-protective ventilation settings and patient synchrony with the ventilator. Papazian *et al.*[61] evaluated the effect of a 2-day course of neuromuscular blocking agents in patients who developed severe ARDS requiring intubation within 48 hours of study enrollment. This randomized, double-blinded trial in 20 multidisciplinary ICUs demonstrated a trend toward improved adjusted 90-day survival and increased time off the ventilator, especially in patients with a PaO_2_/FiO_2_ ratio less than 120.

A systematic review of 33 randomized controlled trials came to the conclusion that the evidence to support pharmacological interventions, namely prostaglandin E1, N-acetylcysteine, the early administration of high dose corticosteroids, or surfactant for ALI and ARDS is insufficient [62,63].

Intermittent prone positioning therapy can improve oxygenation, but has failed to show a survival benefit except as rescue therapy in the severely hypoxemic ARDS patient with P/F ratios of less than 100 (if measured in mm Hg (less than 13 if measured in kPa), but not in patients with P/F ratios of greater than 100 if measured in mm Hg (greater than 13 if measured in kPa) [64-66].

Extracorporeal membrane oxygenation (ECMO) has been used in severe ARDS when the risks of refractory hypoxemia outweigh the risks of this invasive procedure. Although an early NIH study showed a greater volume of blood lost due to systemic coagulation in ECMO patients and no mortality benefit [67], more recent studies have demonstrated improved survival with ECMO in patients following traumatic injury [68,69]. The availability of heparin-bonded circuitry can negate the need for systemic anticoagulation for several days in patients following injury that in the majority of cases is performed with systemic anticoagulation [70]. Newer, mobile, and more compact circuits allow for the use of this life-saving intervention in far-forward military locales and during military transport [71].

Rib fractures are detected in 10% of trauma admissions. In 6% of patients after blunt chest injury, individual ribs are fractured in more than one place and allow paradoxical chest movement with respiration. Surgical management is controversial despite two level one evidence trials favoring operative fixation [72]. Tanaka *et al.*[73] and Granetzny *et al.*[74] reported significantly shorter durations of mechanical ventilation, length of stay in the ICU, and pneumonia with surgical fixation. A retrospective study in 1998 by Voggenreiter [75] had suggested that patients with pulmonary contusions did not benefit from surgical fixation as much as patients without pulmonary contusions did. This trend was also observed in a recent retrospective case-controlled study [76], but did not reach statistical significance. The controversy is further highlighted by two reports of improved pulmonary function testing results after surgical stabilization of flail chest at the 2012 Eastern Association for the Surgery of Trauma annual scientific meeting [77,78]. Large prospective randomized-controlled trials are needed for definite answers to relevant outcome questions.

## Conclusions and recommendations

Acute lung injury and acute respiratory distress syndrome are heterogeneous diseases, the end result of many different types of acute pulmonary injury and with at times overlapping pathogenetic mechanisms [79]. Patients with trauma-associated lung injury have not received as much investigative attention as their medical and sepsis-afflicted counterparts with ALI/ARDS. High-level evidence to recommend ALI/ARDS management strategies tailored to this particular patient population is insufficient at this time.

Although there are several management strategies to improve oxygenation in patients on mechanical ventilation, decisions which therapies to use should be guided by meaningful outcome measures, including reduced duration of mechanical ventilation, length of ICU stay, and mortality [80].

Many trials examining the potential benefits of interventions have used mechanical ventilation strategies that are recognized as harmful today. Until recently, too many studies have failed to compare their intervention group to controls that reflect the current standard of care and mitigate progression of the lung injury. At a minimum, control groups should be ventilated with small tidal volumes as close to 6 ml/kg predicted body weight as patient comfort and ventilator synchrony permit, a minimum PEEP of 5 cm H_2_O, and plateau pressures less than or equal to 30 cm H_2_O per existing guidelines for all patients with ALI/ARDS [81]. While we await future trials, we as clinicians can incorporate what has been shown to save lives a decade ago: lung protective ventilation.

## Competing interests

The authors declare that they have no competing interests.

## Authors' contributions

MB and BB contributed to the literature reviews, manuscript composition and editing. MM contributed to manuscript composition, editing and was responsible for the final product. All authors have read and approved the final manuscript.
